# Aligning perspectives: towards a standardized concept of “complexity” in thyroid surgery. An international web-based survey

**DOI:** 10.1007/s13304-025-02470-0

**Published:** 2025-12-22

**Authors:** Giacomo Di Filippo, Gian Luigi Canu, Leonardo Rossi, Fabio Medas, Federico Cappellacci, Piermarco Papini, Mattia Cammarata, Eleonora Morelli, Giovanni Lazzari, Dorin Serbusca, Alessandro Pasculli, Francesco Paolo Prete, Giuliana Rachele Puglisi, Alessandro Monaco, Luigi Ragucci, Giovanni Cozzolino, Eleonora Lori, Francesco Pennestrì, Pierpaolo Gallucci, Carmela De Crea, Salvatore Sorrenti, Giovanni Docimo, Mario Testini, Marco Raffaelli, Gabriele Materazzi, Pietro Giorgio Calò, The Aligning Perspectives Collaborative Group, Giulia Gobbo, Claudia Bonifazi, Enrico Battistella, Pierpaolo Di Lascio, Marco Puccini, Gennaro Lupone, Francesca Palma, Giovanna Di Meo, Giovanna Pavone, Maurizio Iacobone, Paolo Del Rio, Andrea Borasi, Thea Pierdomenico, Nicola Tartaglia, Luigi Oragano, Antonio Antonino, Gian Luca Ansaldo, Graziano Longo, Maria Ida Amabile, Alessio Giordano, Antonio Toniato, Giorgio Giraudo, Costanza Chiapponi, Gianlorenzo Dionigi, Mustafa Yener Uzunoglu, Omer Yalkin, Agata Dukaczewska, Francesco Pedicini, Simone Beretta, Manuel Felices, Guldeniz Karadeniz Cakmak, Ahmed Mohammed Obeidat, Juan Duenas, Theodora Margariti, Jordi Vidal Fortuny, Ioannis Christakis, Brendan Stack, Donatella Schiavone, Bojan Kovacevic, Angeliki Chorti, Marco Moretti, Gael Guian, Barbara Mullineris, Charles De Ponthaud, Sebastien Gaujoux, Sharjeel Paul, Mechteld de Jong, Agnieszka Dworzyńska, Andreas Muth, Bandar Alharthi, Elena Bonati, Gaurav Agarwal, Paolo Usai, François Ansart, Elena Adelina Toma, Loredana De Pasquale, David Thorsteinsson, Octavian Enciu, Francisco De Santos Iglesias, Sami Abd Elwahab, Giancarlo Basili, Mario Pacilli, Antonio Ambrosi, Trong Anh Nguyen, Andrea Valer Gatti, Milan Jovanovic, Volker Fendrich, Cristina Martinez-Santos, Enzo Bonadies, Rogeh Habashi, Juan Bernar De Oriol, Anurag Tiwary, Tariq Madkhali, Eleftherios Spartalis, Agostino Fernicola, Christian Camenzuli, Andrea Costantino, Han Boon Oh, Tiffany Gan, Sofia Rozani, Somprakas Basu, Marco Palucci, Elissavet Anestiadou, Courtney Gibson, Riccardo Morandi, Pasquale Cianci, Maria Luisa Altana, Akif Enes Arikan, Claudio Casella, Pier Francesco Alesina, Ilia Patrizia Pisano, Martina Mogl, Wah Yang, Ahmet Cem Dural, Michael Stechman, Eva Brugger, Stephan Kersting, Özer Makay, Frank Weber, Eveline Slotema, Johannes Doerner, Gianluca Donatini, Kiyomi Horiuchi, Ludovico Sehnem, Ali Naddaf, Erick Gonzales Laguado, Theodosios Papavramidis, Haythem Najah, Mehmet Ilker Thuran, Jordi Girones, Michele Minuto, Andrea Goldmann, Benedetto Calì, Andreas Zielke, Adela Valdazo Gomez, Teresa Cereser, Jesús María de Villar, Michal Kusinski, Marc Goebel, Sabaretnam Mayilvaganan, Serkan Sari, Sezer Akbulut, Mikhail Bolgov, Jin Wook Yi, Sohail Bakkar, Selen Soylu, Lucia Amorim, Klaas Van Den Heede, Luca Sessa, Anton Engelsman, Francesco Giudici, Radu Mihai, Dieter Morales-Garcia, Vasilis Constantinides, Katrin Brauckhoff, Tugba Matlim Özel, Nikolaos Roukounakis, Aykut Çelik, Göran Wallin, Marcela Linhartová, Yasser Obadiel, Nikolaos Voloudakis, Neil Sharma, Muhammer Ergenç, Frederic Triponez, Maryan Ostafiychuk, Fausto Palazzo, Ioannis Massalis, Georgi Popivanov, Sam Van Slycke, Anislav Gabarski, Zenon Narbuts, Filipe Sá Santos, Martha Trujillo, Muharrem Oner, Claudia Armellin, Andrzej Hellmann, Nikola Slijepcevic, Augustas Beisa, Laurent Brunaud, Max Schneider, Tobias Zingg, Camille Marciniak, Wilhelmina Conradie, Angela Juliane Berger, Andrea De Palma, Damiano Chiari, Inga- Lena Nilsson, Ifongo Bombil, Arian Mokhtari, Samir Jabbar, Mauricio Sierra Salazar, Mehmet Uludag, Nurcihan Aygün, Ozan Caliskan, Mehmet Taner Ünlü, Marie-Laure Matthey Gié, Hunadi Molabe, Mehmet Haciyanli, Huseyin Yuce Bircan

**Affiliations:** 1 Endocrine Surgery Unit, Department of Surgery and Oncology, Verona University Hospital, 37124 Verona, Italy; 2 Department of Surgical Sciences, University of Cagliari, 09042 Monserrato, Italy; 3 Endocrine Surgery Unit, University Hospital of Pisa, 56100 Pisa, Italy; 4 Department of Precision and Regenerative Medicine and Ionian Area, University of Bari, Bari, Italy; 5 Unit of Thyroid Surgery, University of Campania “Luigi Vanvitelli”, Naples, Italy; 6 Department of Surgery, Sapienza University of Rome, Rome, Italy; 7 U.O.C Chirurgia Endocrina E Metabolica, Fondazione Policlinico Universitario Agostino Gemelli IRCCS, Rome, Italy; 8 Centro Di Ricerca in Chirurgia Delle Ghiandole Endocrine E Dell’Obesità (C.R.E.O.), Università Cattolica del Sacro Cuore, Rome, Italy; 9 U.O.C Chirurgia Endocrina, Gemelli Isola - Ospedale Isola Tiberina, Rome, Italy

**Keywords:** Complexity, Thyroidectomy, Complications, Endpoints, Survey

## Abstract

**Introduction:**

Complication rates after thyroidectomy vary widely among centres. Various factors can affect the “complexity” of a case. However, an internationally agreed upon definition of what constitutes a “complex” case in thyroid surgery is currently lacking. We aimed to establish a framework supporting the development of a standardized definition of “complexity” in thyroid surgery by collecting endocrine surgeons’ opinions through a survey.

**Materials and methods:**

A 28-item survey was distributed through the mailing lists of the Italian and European Societies of Endocrine Surgeons and via social media. Questions explored respondents’ opinions on determinants and endpoint measures of “complexity”. Responses were compared by unit and individual thyroidectomy volume (> 50 vs. < 50 cases/year), and by routine use of pre-operative ultrasound and intra-operative nerve monitoring.

**Results:**

Among 192 respondents, 97.3% acknowledged the potential usefulness of a shared definition of “complexity” in thyroid surgery for patients’ workflow optimization.

Permanent vocal-cord palsy (78.6%), operative duration (77.1%) and permanent hypoparathyroidism (77.1%) were most frequently chosen as appropriate endpoint measures of “complexity”. Among determinants, previous neck surgery, adhesions/infiltration, mediastinal extension and large thyroid volume were considered impactful by the majority of respondents. High volume surgeons more frequently selected permanent palsy, tracheal injury and R1 margins as endpoints, and BMI as determinants of “complexity” (all *P*s ≤ 0.05).

**Conclusion:**

Endocrine surgeons recognize the need for a standardized definition of “complexity” in thyroid surgery to enhance risk stratification and care. Perceived complexity varies with proficiency. Collected data support a reproducible framework, to be validated in future studies.

**Supplementary Information:**

The online version contains supplementary material available at 10.1007/s13304-025-02470-0.

## Background

Thyroidectomy is one of the most frequently performed endocrine surgeries worldwide, with more than 35,000 procedures undertaken each year in Italy alone [1]. Surgical outcomes and complication rates vary widely among centres [2–6]. Previous studies have suggested both patient‑ and disease‑specific factors as possible contributors to this variability [7–12]. For instance, Mekel et al. [13] identified older age, male gender and a higher burden of comorbidities as independent predictors of postoperative adverse events, with octogenarians experiencing the greatest risk, while Karabeyoǧlu et al. [14] demonstrated that large gland volumes increase the likelihood of recurrent laryngeal nerve injury and peri‑operative haemorrhage. Other studies reported an increased risk of complications following surgery for large and/or retrosternal goiters, autoimmune thyroid disease, or thyroid cancer [10, 15–17].

Although technical difficulty and patient frailty undoubtedly heighten the likelihood of adverse events [18, 19], a common finding in many surgical settings [20, 21], compelling evidence [22–24] shows that greater surgical experience and higher procedural volumes can mitigate these risks, yielding superior outcomes and reduced healthcare costs.

These observations indicate that postoperative complication rate may be influenced by multiple factors, often collectively referred to in clinical practice as case “complexity”, which in turn may be modulated to a certain degree by the operator’s proficiency. Indeed, “complexity” in thyroid surgery arises from the interplay between patient characteristics, histo-pathological factors, and environmental considerations such as instrument availability or surgical setting [25]. However, in the absence of an internationally agreed-upon definition clarifying its determinants and how to measure it, “complexity” will remain a subjective concept, heavily influenced by individual experience and technical proficiency. Conversely, a standardised definition would enable reliable risk stratification, support appropriate referral pathways, optimize peri‑operative planning and validate meaningful audit and quality‑improvement initiatives.

Similar efforts in other surgical specialties illustrate the value of such an approach. Kawaguchi et al. [26] created a “difficulty score” for laparoscopic liver resection that predicts morbidity and mortality, while Farshad et al. [27] proposed Spine Surgical “complexity” Categories that correlate with operative time, blood loss and length of stay.

In one of the first attempts to standardise “complexity” in thyroid surgery, Schneider et al. [28] introduced the Thyroidectomy Difficulty Scale, a four-item intra-operative score that assesses thyroid vascularity, friability, mobility and size, and correlates the total score with operative time and complication risk. However, as a subjective tool that relies only on intra-operative data, this scale has little use for pre-operative risk stratification and peri-operative planning.

To address this gap in endocrine surgery, a three‑phase protocol was conceived, which involved: (1) an international survey to collect the opinions of international endocrine surgeons on which endpoint measures characterize a “complex” thyroidectomy case and the determinants thereof; (2) a prospective observational study to create and validate a predictive model for postoperative complications based on the endpoints suggested by the majority of survey respondents; and (3) a modified Delphi consensus to establish an expert-endorsed, standardized definition of “complexity” in thyroid surgery. The present paper reports the results of Phase 1, and lays the groundwork for the subsequent analytical and consensus phases.

## Methods

On July 1st, 2024, the United Italian Society of Endocrine Surgery (SIUEC, Società Italiana Unitaria di Endocrinochirurgia) nominated a Steering Committee including 5 experienced endocrine surgeons with the aim to conduct a cross-sectional survey among international endocrine surgeons on the concept of “complexity” in thyroid surgery.

The survey was developed and reported as per the Checklist for Reporting Results of Internet E-Surveys (CHERRIES) [29]. Formal ethical approval was not needed, as the survey involved voluntary, non-incentivized participation, with anonymously collected data.

### Survey development and distribution

The survey (https://forms.gle/h5VtWuQfcjezq8eD6) was developed on the Google Form platform (Alphabet inc., Mountain View, California, USA). The Steering Committee agreed on the items to be included in the survey after 3 rounds of emails, considering both their relevance and clarity. The survey was developed in English to ensure broad dissemination among an international audience of respondents.

The survey comprised 2 sections. The first section investigated respondents’ baseline characteristics, such as country of practice, proficiency, and surgical setting. The second section collected respondents’ opinions on the concept of “complexity” in thyroid surgery by exploring their degree of agreement regarding the relevance of specific endpoint measures and determinants of “complexity” in open, minimally invasive and remote access thyroidectomy.

The survey was made available online from September 1st, 2024 to November 15th, 2024. The survey was distributed among surgeons through the mailing lists of SIUEC and European Society of Endocrine Surgeons (ESES), and through a social media platform (LinkedIn, LinkedIn Corporation). A reminder email was sent on October 1st, 2024.

Eligible participants were endocrine surgeons (general surgeons, ENT) or residents and fellows with a particular interest in endocrine surgery.

Unit proficiency was defined as a surgical unit performing > 50 thyroidectomies/year while personal proficiency was defined as a surgeon performing  > 50 thyroidectomies/year [30]. Survey responses were stratified and compared across the following dichotomous categories: (1) unit proficiency (> 50 vs. < 50 thyroidectomies/year), (2) individual surgeon proficiency (> 50 vs. < 50 thyroidectomies/year), (3) routine personal use of pre-operative ultrasound (US) (yes vs. no), and (4) routine use of intra-operative nerve monitoring (yes vs. no). Overall Agreement (OA) was obtained through the sum of percentages of respondents who somewhat or strongly agreed with the proposed item. Likewise, Overall Disagreement (OD) was obtained through the sum of percentages of respondents who somewhat or strongly disagreed with the proposed item. Permanent hypoparathyroidism and vocal cord palsy were specified as conditions persisting for more than 6 months after surgery.

### Statistical analysis

After data extraction, duplicate entries (i.e., multiple responses from the same participant) were manually sought and discarded.

Categorical variables were expressed as absolute numbers and relative percentages. Continuous variables were reported as medians and interquartile ranges. Differences between groups were tested using chi square analysis for dichotomous variables while a Mann Whitney test was employed for ordinal data (Likert scale data).

A *p*-value < 0.05 was considered as statistically significant.

Data analysis was performed on SPSS version 25.0 (IBM Corp., Armonk, New York).

## Results

**Fig. 1:**
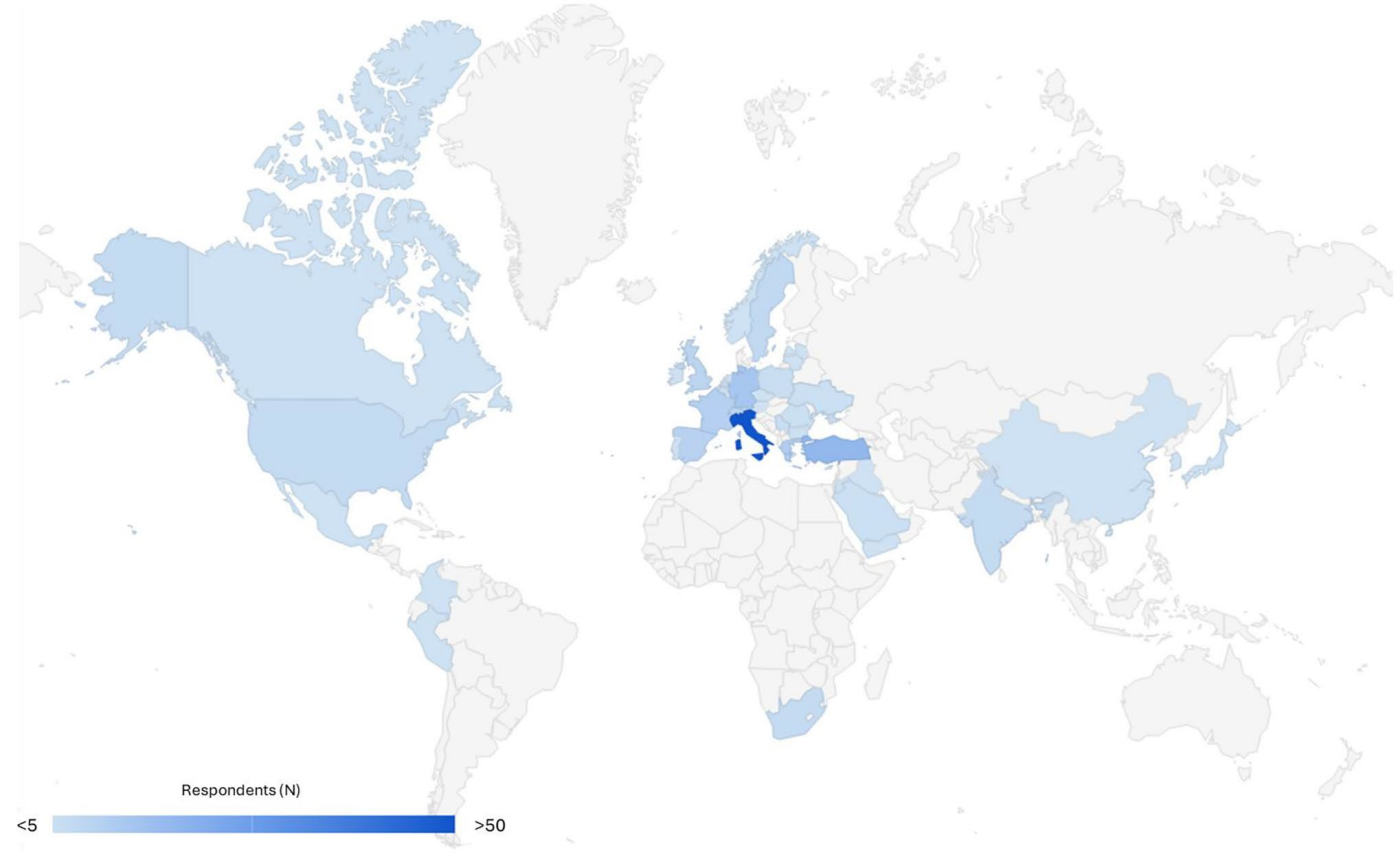
Respondents’ geographical distribution

A total of 192 international surgeons participated in the survey. Most of the respondents were based in European Countries (85.4%), with a substantial participation from Italy (32.3%). The geographical distribution of the respondents is illustrated in Fig. 1.

Table 1 summarizes respondents’ answers to the first section of the survey.

**Table 1:** Respondents’ baseline characteristics

		N (%)
Which surgical specialty are you specialized in?	ENT	4 (2.1)
	General surgery	180 (93.8)
	General surgery, ENT	5 (2.6)
	General surgery, thoracic surgery	3 (1.6)
How long have you been working as a surgeon in your field?	I’m in residency	9 (4.7)
	< 5 years	18 (9.4)
	5–10 years	47 (24.5)
	> 10 years	118 (61.5)
In which of the following setting are you working?	Affiliated private hospital*	25 (13)
	Private practice	4 (2.1)
	Public hospital–non teaching	12 (6.3)
	Public hospital–teaching	151 (78.6)
How many total thyroidectomies does your unit perform YEARLY?	< 20	3 (1.6)
	20–49	18 (9.4)
	50–200	69 (35.9)
	> 200	102 (53.1)
How many total thyroidectomies do you personally perform YEARLY?	< 20	21 (10.9)
	20–49	38 (19.8)
	50–200	96 (50)
	> 200	37 (19.3)
Do you personally and routinely use ultrasound to evaluate your patient before surgery?	No	60 (31.3)
	Yes	132 (68.8)
Does your unit routinely use intraoperative nerve monitoring while performing thyroidectomies?	No	46 (24)
	Yes	146 (76)
Does your unit routinely use parathyroid autofluorescence technology while performing thyroidectomies?	No	157 (81.8)
	Yes	35 (18.2)
Do you routinely use advanced hemostasis devices (e.g., ligasure, harmonic scalpel, etc..) while performing thyroidectomies?	No	28 (14.6)
	Yes	164 (85.4)
Do you routinely use topical hemostatic agents (e.g., tabotamp, tachosil, hemopatch, …) while performing thyroidectomies?	No	101 (52.6)
	Yes	91 (47.4)
Do you believe a standardized definition of “complexity” in thyroid surgery would be useful in stratifying the patient’s baseline risk of postoperative complications and therefore selecting the best workflow to reduce said risk both in open surgery and minimally invasive/remote access surgery?	No	5 (2.6)
	Yes	187 (97.4)

*ENT* otolaryngologist, *IONM* intraoperative nerve monitoring, *ICU* intensive care unit

*Private hospitals that have an agreement with the national health service (NHS) to provide services that can be reimbursed by the NHS

**Fig. 2:**
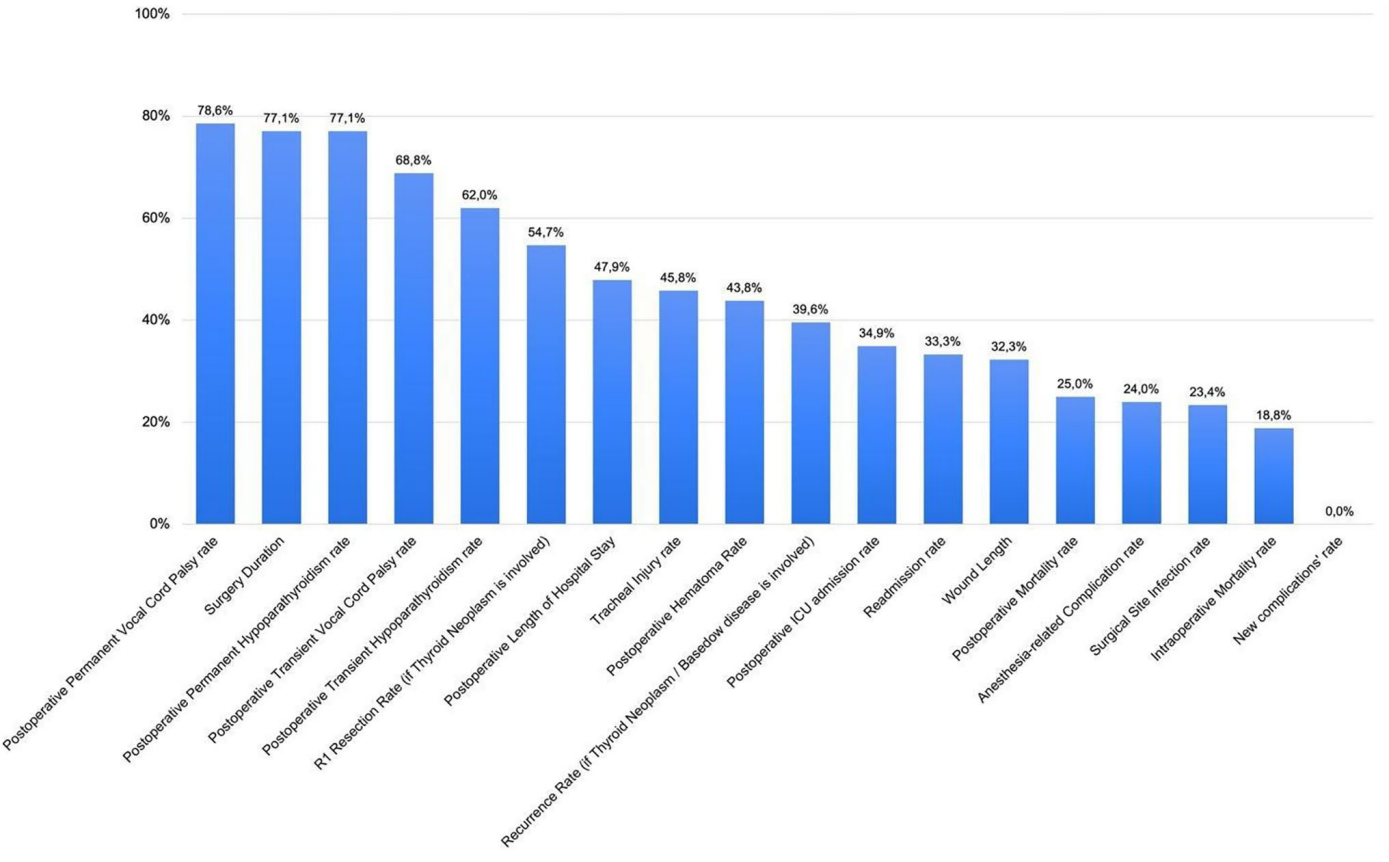
Distribution of selected endpoints among respondents. ICU, intensive care unit

When respondents were asked which endpoint measures they believed would be impacted by a “Complex Thyroidectomy” (Fig. 2), the most frequently selected ones were “Postoperative permanent vocal cord palsy rate” (78.6%), “Surgery duration” (77.1%), and “Postoperative permanent hypoparathyroidism rate” (77.1%). Less frequently selected endpoints included “Intraoperative mortality rate” (18.8%), “Surgical site infection rate” (23.4%), and “Anesthesia-related complication rate” (24%).

**Fig. 3:**
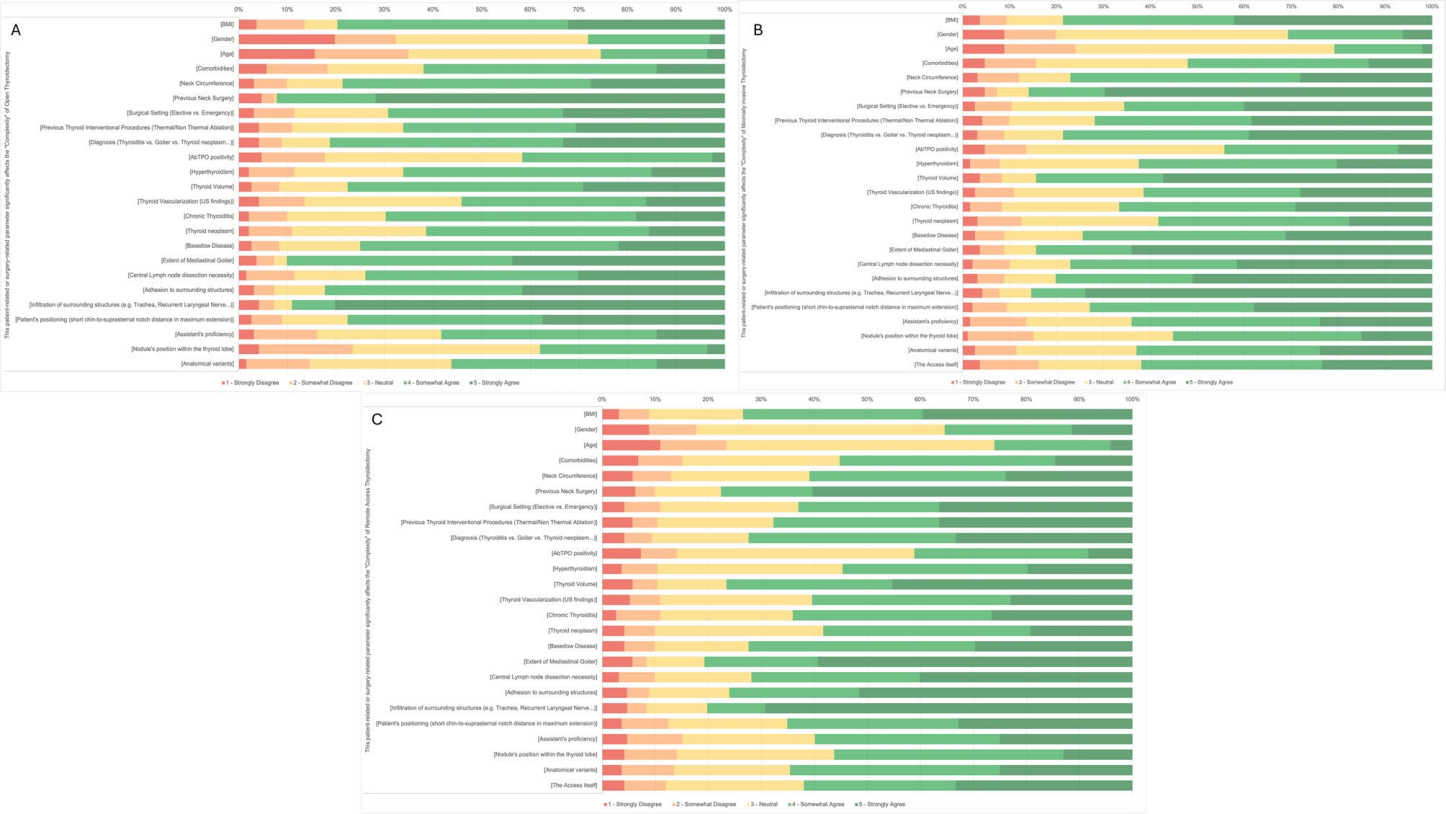
Respondents’ degree of agreement regarding the potential impact of specific patient- or surgery-related parameters on the “complexity” of thyroidectomies. **a**: determinants of complexity for open thyroidectomy; **b**: determinants of complexity for minimally invasive thyroidectomy; **c**: determinants of complexity for remote access thyroidectomy. BMI, body mass index; AbTPO, anti-thyroperoxidase antibodies; US, ultrasound

In the following items, participants were asked to rate on a 5-point scale their degree of agreement (1 = Strongly disagree; 5 = Strongly agree) regarding the potential impact of specific patient- or surgery-related parameters on the “complexity” of thyroidectomies, divided by surgical access. Among factors potentially influencing the perceived “complexity” of open thyroidectomy (Fig. 3A), previous neck surgery and infiltration of adjacent structures were widely regarded as contributors. Regarding minimally invasive thyroidectomy (Fig. 3B), agreed upon determinants included previous neck surgery, thyroid volume, extent of mediastinal goiter, infiltration of surrounding structures, and adhesion to surrounding tissues. Regarding remote access thyroidectomy (Fig. 3C), the variables most frequently agreed upon were infiltration of surrounding structures, adhesion to surrounding structures, extent of goiter, and previous neck surgery. Conversely, respondents less frequently considered age, gender and anti-thyroid peroxidase (AbTPO) antibody positivity to significantly affect the complexity of a thyroidectomy across all surgical approaches.

**Fig. 4:**
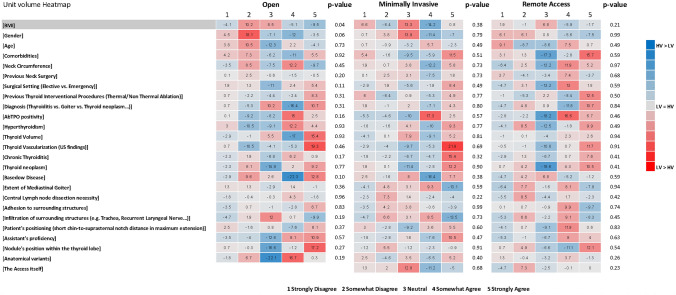
Unit volume heatmap. The numbers in each cell represent the result of the following formula (low volume group %)–(high volume group %). BMI, body mass index; AbTPO, anti-thyroperoxidase antibodies; LV, low volume; HV, high volume

Surgeons working in surgical units performing more than 50 thyroidectomies per year identified the incidence of permanent vocal cord palsy (80.7% vs. 61.9%, *p* = 0.05), tracheal injury (48.5% vs. 23.8%, *p* = 0.03), and R1 resections in oncologic cases (57.3% vs. 33.3%, *p* = 0.04) as endpoint measures more frequently than surgeons working in lower-volume surgical units. Furthermore, they considered BMI a significant determinant of the “omplexity” of open thyroidectomy more frequently compared to surgeons with lower unit proficiency (OA 81.3% vs. 66.7%, *p* = 0.04) (Supplementary Table 1; Fig. 4).

Surgeons with a case volume > 50 thyroidectomies per year reported a greater routine use of intraoperative nerve monitoring (81.2% vs. 64.4%, *p* = 0.01) and less use of topical hemostatic agents (42.1% vs. 59.3%, *p* = 0.03) compared to surgeons with a case volume lower than 50 thyroidectomies per year. Additionally, the former more frequently reported the rate of R1 resections in case of malignancy as an endpoint measure compared to the latter (59.4% vs. 44.1%, *p* = 0.05). With regards to the determinants of “complexity”, surgeons with higher case volume agreed to infiltration of surrounding structures being a significant factor for both open and minimally invasive thyroidectomy more frequently than surgeons with lower case volume (OA 94% vs. 78%, *p* = 0.01).

**Fig. 5:**
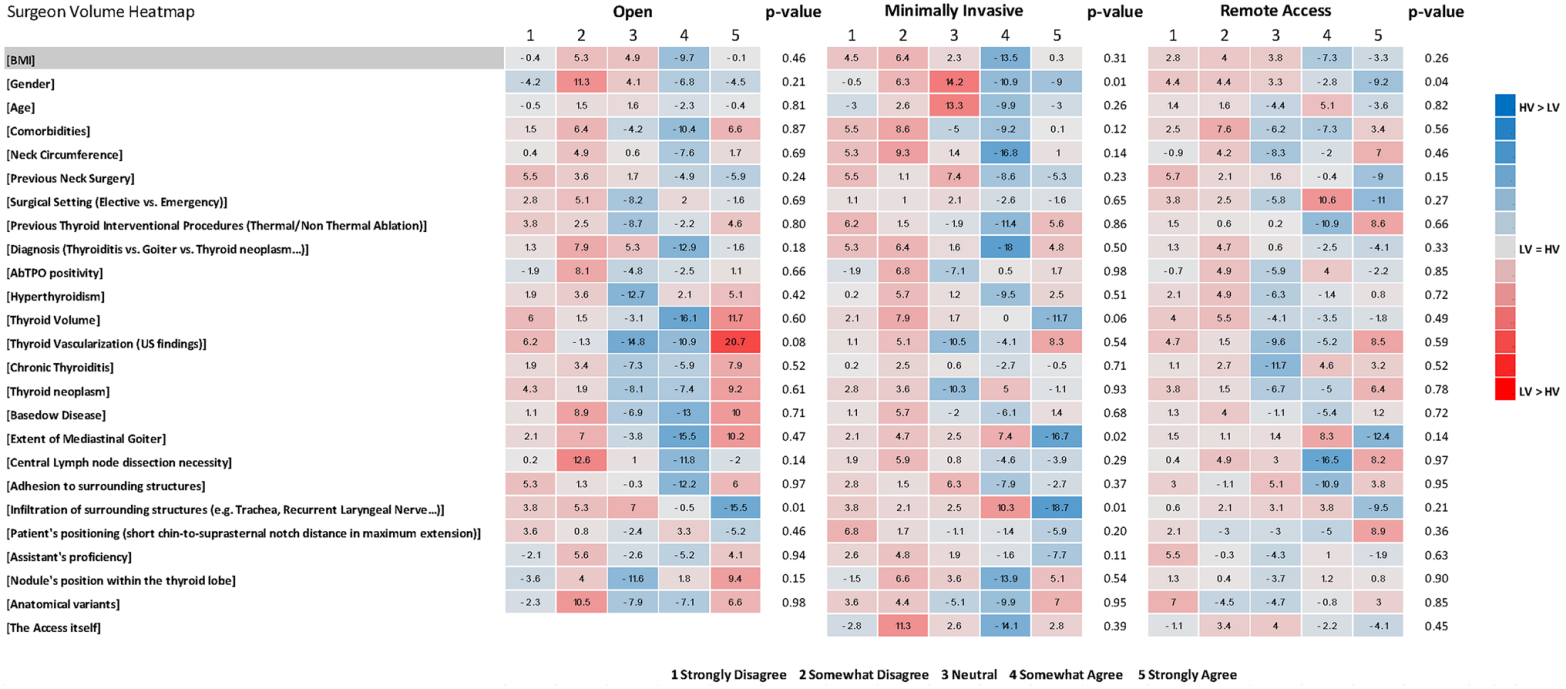
Surgeon volume heatmap. The numbers in each cell represent the result of the following formula (low volume group %)–(high volume group %). BMI, body mass index; AbTPO, anti-thyroperoxidase antibodies; US, ultrasound; LV, low volume; HV, high volume

Additionally, respondents with higher case volume considered gender (OA 36.8% vs. 16.9%, *p* = 0.01) and extent of goiter (OA 87.2% vs. 77.9%, *p* = 0.02) as determinants of increased “complexity” in minimally invasive thyroidectomy more frequently than respondents with lower case volume. Surgeons with higher case volume perceived gender as influencing the “complexity” of remote access thyroidectomy more frequently than surgeons with lower personal proficiency (OA 39.1% vs. 27.1%, *p* = 0.04). Notably, a significant difference in terms of respondents’ opinion on the potential usefulness of a shared definition of “complexity” emerged (90.5% vs. 98.2%, *p* = 0.04). (Supplementary Table 2; Fig. 5).

**Fig. 6:**
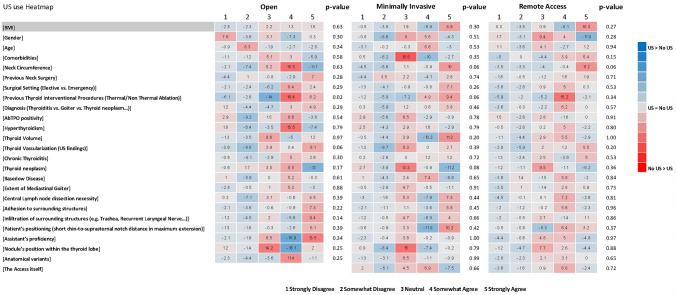
Ultrasound use heatmap. The numbers in each cell represent the result of the following formula (No US group %)–(US group %). BMI, body mass index; AbTPO, anti-thyroperoxidase antibodies; US, ultrasound

Surgeons who don’t routinely perform preoperative US assessments, considered a history of thyroid interventional procedure to significantly increase the “complexity” of open thyroidectomy more frequently than those who routinely use US (OA 81.7% vs. 59.1%, *p* = 0.02) (Supplementary Table 3; Fig. 6).

**Fig. 7:**
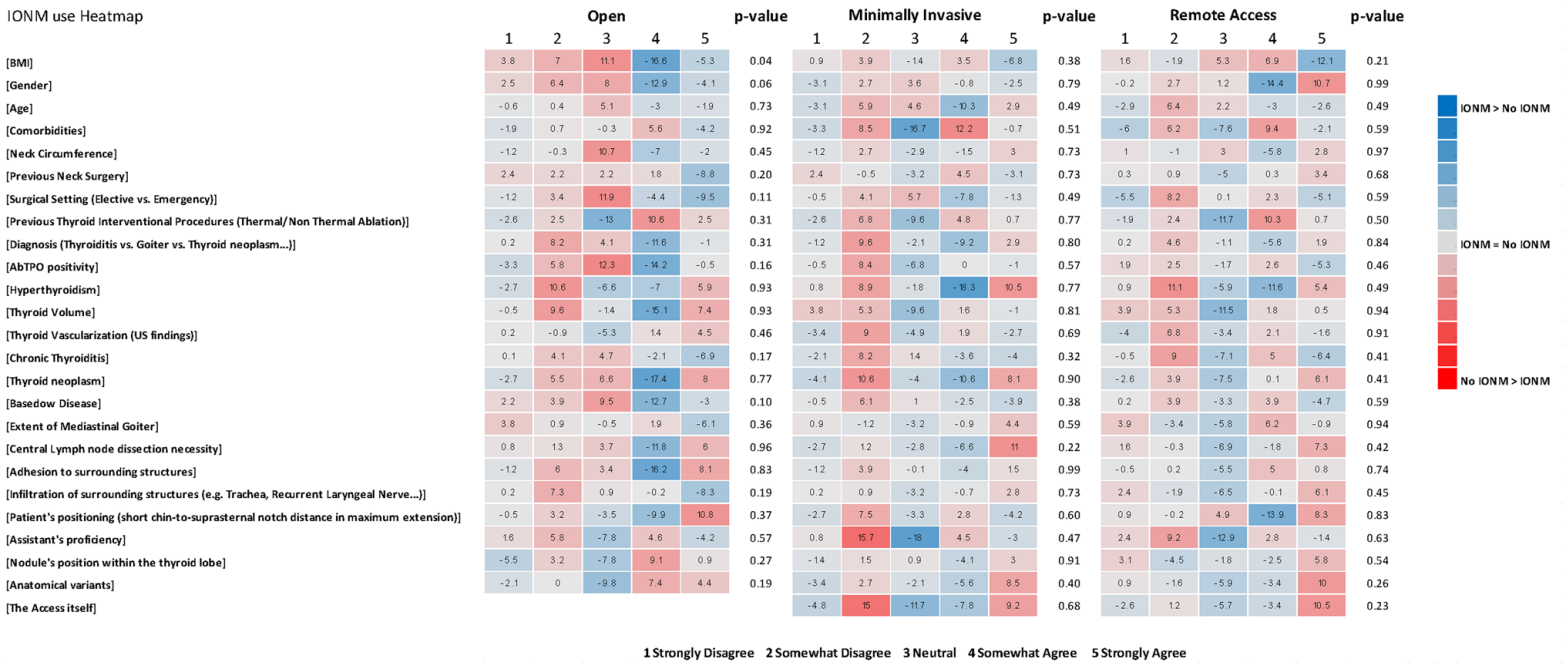
7 IONM use heatmap. The numbers in each cell represent the result of the following formula (No IONM group %)–(IONM group %). BMI, body mass index; AbTPO, anti-thyroperoxidase antibodies; IONM, intraoperative nerve monitoring; US, ultrasound

Surgeons who routinely use IONM, also use advanced hemostatic devices more frequently than non-routine IONM users (88.4% vs. 76.1%, *p* = 0.04). They also identified the rate of permanent vocal cord palsy (82.2% vs. 67.4%, *p* = 0.03) and permanent hypoparathyroidism (80.8% vs. 65.2%, *p* = 0.03) as appropriate endpoint measures more frequently compared to non-routine users. BMI was considered a determinant of “complexity” of open thyroidectomy by routine IONM users more frequently than non-routine users (OA 85% vs. 63.1%, *p* = 0.04) (Supplementary Table 4; Fig. 7).

## Discussion

This survey represents the first attempt to capture how endocrine surgeons worldwide conceptualise “complexity” in thyroid surgery, and which outcomes they believe are the most sensitive to it. Previous studies [4, 7, 16, 31–33] have catalogued an extensive array of patient- and disease-specific variables that correlate with postoperative morbidity, yet the surgeon’s subjective perception of “complexity”, and the way that perception is modulated by experience, has remained largely unexplored so far. Interestingly, the vast majority of respondents indicated that having a shared, standardized, definition of “complexity” would be useful in their clinical practice. This underscores both the timeliness of this study and the relevance of the forthcoming steps in developing a standardized framework for defining “complexity” in thyroid surgery.

The majority of respondents were high-volume general surgeons working in high-volume, public teaching hospitals. Two thirds of respondents reported routinely using preoperative US evaluation and IONM, while only 20% reported using intraoperative autofluorescence devices. These findings are in line with the results from the survey by Ritter et al. [34], who found that 72% of responding surgeons routinely used IONM. Conversely, US pre-operative evaluation resulted more common among the surgeons interviewed in our study compared to those interviewed by Maniakas et al. [35].

Across all three surgical approaches (i.e., open, minimally invasive, and remote access) a relatively high proportion of respondents dismissed age, gender and AbTPO positivity as meaningful determinants of thyroidectomy “complexity”, while consistently emphasizing the importance of prior neck surgery and local anatomic challenges. For open procedures, infiltration or adhesions of adjacent structures and previous surgery were most commonly chosen as determinants; for minimally invasive cases, large gland volume; and for remote-access techniques, infiltration, gland extension, adhesions and prior surgery were most frequently reported as influential.

These findings highlight a clear consensus that thyroidectomy “complexity” is driven primarily by technical and anatomical factors, rather than patient demographics or autoimmune serology. This is somewhat in contrast with current literature, which has so far associated thyroidectomy difficulty with male gender, young age, patient’s BMI, and AbTPO positivity (mostly employed as a surrogate of thyroiditis) [7, 36]. This result may be attributed to the fact that the majority of our respondents were proficient surgeons, whose clinical experience likely mitigates the impact of these variables on perceived surgical “complexity”.

Conversely, the high degree of agreement on the importance of adhesions, infiltration, and gland volume and extension as contributors to “complexity” is consistent with findings by Prete et al. and Schneider et al. [28, 37], among others, who underscored a strong association between these factors and longer operative times and postoperative complications.

In our survey, when asked which outcomes most likely result from complex thyroidectomy cases, surgeons most frequently selected permanent recurrent laryngeal nerve palsy, prolonged operative time, and permanent hypoparathyroidism. Although not unanimous, there was broad consensus on these as the most relevant endpoint measures of surgical “complexity”.

Operative duration has been frequently identified in the literature as both a marker and a measure of thyroidectomy “complexity” [36–40]. Several reports even define surgical “complexity” based on increased operative time [7, 34]. Among others, Dong et al. [7] developed a predictive model for difficult thyroidectomy using surgery duration above the 75th percentile as a threshold. Notably, this model also correlated with a higher risk of postoperative complications, which may suggest a potential link between prolonged operative time and adverse surgical outcomes.

However, as reported in the literature [7, 36], longer surgery duration and higher complication rates share many overlapping predictors. This collinearity may suggest that they represent different expressions of the same underlying surgical “complexity”.

Interestingly, some studies have reported a mismatch between the risk factors associated with difficult thyroidectomy defined by operative duration and those linked to postoperative complications [34, 36].

While it is reasonable to assume that truly complex cases inherently demand more time and effort to complete the necessary surgical maneuvers, the fact that operative time is heavily influenced by individual factors such as surgeon fatigue, team dynamics, and institutional efficiency, limit its reliability as a standalone indicator of “complexity”. A comprehensive, multiparametric measure that incorporates a range of factors beyond operative time and complications is therefore needed. This approach is supported by our respondents, who did not point to a single endpoint measure of thyroidectomy “complexity”, but rather identified multiple relevant endpoints.

Surgeon experience significantly affects both operative time and complication rates, alongside other variables (e.g., costs, oncological results) [22, 23]. In our survey, working in a high-volume center and being a high-volume surgeon, alongside routinely using US and IONM, appeared to modulate both the selection of relevant endpoints and the perception of factors contributing to surgical “complexity”. In a study by Sosa et al. [22], later confirmed by subsequent research [23, 24], length of stay, complication rates and healthcare costs after thyroidectomy were associated with surgeon’s proficiency, even after adjustment for case mix and hospital volume. In line with these findings and after extensive review of the literature, the ESES positional statement [30] concluded that higher surgeon volume mitigates the excess morbidity associated with autoimmune thyroid disease and thyroid cancer, and is associated with better oncologic results, justifying their preferential referral to high-volume endocrine surgeons. Accordingly, integrating surgeon experience within a predicted “complexity” workflow would align case allocation with surgeon’s proficiency and promote safer, more efficient care.

Collectively, the results from the present survey highlight several key aspects of the concept of surgical “complexity” in thyroid surgery. First, “complexity” likely arises from a dynamic interplay between patient-specific factors, disease characteristics, the availability and use of technological adjuncts, and, perhaps most importantly, the experience of the operating surgeon. Prolonged operative time and the occurrence of thyroidectomy-specific complications represent the most intuitive and commonly recognized endpoints reflecting this interplay, but they are not the only ones. The variability of responses across different levels of proficiency emerged from our survey underscores the need for a standardized, multidimensional framework for defining surgical “complexity” in thyroid surgery, by integrating anatomical considerations, patient-specific factors, and surgical experience. This could ultimately support the development of a composite “complexity” predictive model, which should be further investigated for its associations with clinically relevant outcomes. Such a model would have wide-reaching implications. Preoperatively, it would enable more accurate risk stratification, resource allocation, and patient counseling. In surgical training, it would support the alignment of case difficulty with trainee competency. Furthermore, it would facilitate inter-surgeon and inter-institutional comparisons by adjusting outcomes based on the underlying “complexity” of cases. This, in turn, could inform quality improvement initiatives aimed at optimizing patient workflow at both the institutional and national level.

This study has the following main limitations. First, as an online survey, it was inherently prone to coverage bias, potentially excluding surgeons with limited access to or engagement with digital platforms. This recruitment strategy may also partly explain the relatively small sample size. Secondly, the voluntary nature of participation may have introduced a self-selection bias, likely favoring surgeons with a particular interest in the subject or well-established opinions on the topic. Furthermore, we cannot exclude the potential influence of social desirability bias, which is inherent to self-reported data, as respondents may be more inclined to endorse knowledge that aligns with current literature. Finally, the respondent pool was predominantly composed of high-volume, academically affiliated endocrine surgeons, resulting in a sampling skew. As a result, the responses to the survey likely reflect the perspectives and practices of a specific subset of surgeons and may not accurately represent broader real-world practices or views, thus present findings should be interpreted with caution.

## Conclusions

The results of the present survey highlight that endocrine surgeons recognize the need for a standardized definition of “complexity” in thyroid surgery, underscoring the clinical importance of preoperative “complexity” assessment for improved risk stratification and optimized patient care. Notably, the perception of “complexity” appears to vary with the surgeon’s experience and practice context. These findings could provide foundational data for developing a shared, reproducible “complexity” model tailored to thyroid surgery, which could guide surgical planning and promote outcome optimization. However, given the survey-based design and its inherent limitations, any model derived from these results will need to be developed through prospective, ideally multicenter studies with external validation before influencing clinical practice.

## Supplementary Information

Below is the link to the electronic supplementary material.

Supplementary Material 1.

## Data Availability

The datasets generated and analysed during the current study are available from the corresponding author on reasonable request.
