# Correction: Aligning perspectives: towards a standardized concept of “complexity” in thyroid surgery—an international web-based survey

**DOI:** 10.1007/s13304-026-02635-5

**Published:** 2026-03-24

**Authors:** Giacomo Di Filippo, Gian Luigi Canu, Leonardo Rossi, Fabio Medas, Federico Cappellacci, Piermarco Papini, Mattia Cammarata, Eleonora Morelli, Giovanni Lazzari, Dorin Serbusca, Alessandro Pasculli, Francesco Paolo Prete, Giuliana Rachele Puglisi, Alessandro Monaco, Luigi Ragucci, Giovanni Cozzolino, Eleonora Lori, Francesco Pennestrì, Pierpaolo Gallucci, Carmela De Crea, Salvatore Sorrenti, Giovanni Docimo, Mario Testini, Marco Raffaelli, Gabriele Materazzi, Pietro Giorgio Calò, The Aligning Perspectives Collaborative Group, Giulia Gobbo, Claudia Bonifazi, Enrico Battistella, Pierpaolo Di Lascio, Marco Puccini, Gennaro Lupone, Francesca Palma, Giovanna Di Meo, Giovanna Pavone, Maurizio Iacobone, Paolo Del Rio, Andrea Borasi, Thea Pierdomenico, Nicola Tartaglia, Luigi Oragano, Antonio Antonino, Gian Luca Ansaldo, Graziano Longo, Maria Ida Amabile, Alessio Giordano, Antonio Toniato, Giorgio Giraudo, Costanza Chiapponi, Gianlorenzo Dionigi, Mustafa Yener Uzunoglu, Omer Yalkin, Agata Dukaczewska, Francesco Pedicini, Simone Beretta, Manuel Felices, Guldeniz Karadeniz Cakmak, Ahmed Mohammed Obeidat, Juan Duenas, Theodora Margariti, Jordi Vidal Fortuny, Ioannis Christakis, Brendan Stack, Donatella Schiavone, Bojan Kovacevic, Angeliki Chorti, Marco Moretti, Gael Guian, Barbara Mullineris, Charles De Ponthaud, Sebastien Gaujoux, Sharjeel Paul, Mechteld de Jong, Agnieszka Dworzyńska, Andreas Muth, Bandar Alharthi, Elena Bonati, Gaurav Agarwal, Paolo Usai, François Ansart, Elena Adelina Toma, Loredana De Pasquale, David Thorsteinsson, Octavian Enciu, Francisco De Santos Iglesias, Sami Abd Elwahab, Giancarlo Basili, Mario Pacilli, Antonio Ambrosi, Trong Anh Nguyen, Andrea Valer Gatti, Milan Jovanovic, Volker Fendrich, Cristina Martinez-Santos, Enzo Bonadies, Rogeh Habashi, Juan Bernar De Oriol, Anurag Tiwary, Tariq Madkhali, Eleftherios Spartalis, Agostino Fernicola, Christian Camenzuli, Andrea Costantino, Han Boon Oh, Tiffany Gan, Sofia Rozani, Somprakas Basu, Marco Palucci, Elissavet Anestiadou, Courtney Gibson, Riccardo Morandi, Pasquale Cianci, Maria Luisa Altana, Akif Enes Arikan, Claudio Casella, Pier Francesco Alesina, Ilia Patrizia Pisano, Martina Mogl, Wah Yang, Ahmet Cem Dural, Michael Stechman, Eva Brugger, Stephan Kersting, Özer Makay, Frank Weber, Eveline Slotema, Johannes Doerner, Gianluca Donatini, Kiyomi Horiuchi, Ludovico Sehnem, Ali Naddaf, Erick Gonzales Laguado, Theodosios Papavramidis, Haythem Najah, Mehmet Ilker Thuran, Jordi Girones, Michele Minuto, Andrea Goldmann, Benedetto Calì, Andreas Zielke, Adela Valdazo Gomez, Teresa Cereser, Jesús María de Villar, Michal Kusinski, Marc Goebel, Sabaretnam Mayilvaganan, Serkan Sari, Sezer Akbulut, Mikhail Bolgov, Jin Wook Yi, Sohail Bakkar, Selen Soylu, Lucia Amorim, Klaas Van Den Heede, Luca Sessa, Anton Engelsman, Francesco Giudici, Radu Mihai, Dieter Morales-Garcia, Vasilis Constantinides, Katrin Brauckhoff, Tugba Matlim Özel, Nikolaos Roukounakis, Aykut Çelik, Göran Wallin, Marcela Linhartová, Yasser Obadiel, Nikolaos Voloudakis, Neil Sharma, Muhammer Ergenç, Frederic Triponez, Maryan Ostafiychuk, Fausto Palazzo, Ioannis Massalis, Georgi Popivanov, Sam Van Slycke, Anislav Gabarski, Zenon Narbuts, Filipe Sá Santos, Martha Trujillo, Muharrem Oner, Claudia Armellin, Andrzej Hellmann, Nikola Slijepcevic, Augustas Beisa, Laurent Brunaud, Max Schneider, Tobias Zingg, Camille Marciniak, Wilhelmina Conradie, Angela Juliane Berger, Andrea De Palma, Damiano Chiari, Inga- Lena Nilsson, Ifongo Bombil, Arian Mokhtari, Samir Jabbar, Mauricio Sierra Salazar, Mehmet Uludag, Nurcihan Aygün, Ozan Caliskan, Mehmet Taner Ünlü, Marie-Laure Matthey Gié, Hunadi Molabe, Mehmet Haciyanli, Huseyin Yuce Bircan

**Affiliations:** 1 Endocrine Surgery Unit, Department of Surgery and Oncology, Verona University Hospital, 37124 Verona, Italy; 2 Department of Surgical Sciences, University of Cagliari, 09042 Monserrato, Italy; 3 Endocrine Surgery Unit, University Hospital of Pisa, 56100 Pisa, Italy; 4 Department of Precision and Regenerative Medicine and Ionian Area, University of Bari, Bari, Italy; 5 Unit of Thyroid Surgery, University of Campania “Luigi Vanvitelli”, Naples, Italy; 6 Department of Surgery, Sapienza University of Rome, Rome, Italy; 7 U.O.C Chirurgia Endocrina E Metabolica, Fondazione Policlinico Universitario Agostino Gemelli IRCCS, Rome, Italy; 8 Centro Di Ricerca in Chirurgia Delle Ghiandole Endocrine E Dell’Obesità (C.R.E.O.), Università Cattolica del Sacro Cuore, Rome, Italy; 9 U.O.C Chirurgia Endocrina, Gemelli Isola - Ospedale Isola Tiberina, Rome, Italy

** Correction to: Updates in Surgery** 10.1007/s13304-025-02470-0

In this article the author name Papavramidis was incorrectly written as Papavradimis in the collaborative group.

Also, The table 1 legend has been wrongly placed in the middle of the article text.

So, the table 1 legend has been updated correctly.

The updated and un update Table 1 legend as follows:

Uncorrect:

**Table 1:** Respondents’ baseline characteristics

		N (%)
Which surgical specialty are you specialized in?	ENT	4 (2.1)
	General surgery	180 (93.8)
	General surgery, ENT	5 (2.6)
	General surgery, thoracic surgery	3 (1.6)
How long have you been working as a surgeon in your field?	I’m in residency	9 (4.7)
	< 5 years	18 (9.4)
	5–10 years	47 (24.5)
	> 10 years	118 (61.5)
In which of the following setting are you working?	Affiliated private hospital*	25 (13)
	Private practice	4 (2.1)
	Public hospital–non teaching	12 (6.3)
	Public hospital–teaching	151 (78.6)
How many total thyroidectomies does your unit perform YEARLY?	< 20	3 (1.6)
	20–49	18 (9.4)
	50–200	69 (35.9)
	> 200	102 (53.1)
How many total thyroidectomies do you personally perform YEARLY?	< 20	21 (10.9)
	20–49	38 (19.8)
	50–200	96 (50)
	> 200	37 (19.3)
Do you personally and routinely use ultrasound to evaluate your patient before surgery?	No	60 (31.3)
	Yes	132 (68.8)
Does your unit routinely use intraoperative nerve monitoring while performing thyroidectomies?	No	46 (24)
	Yes	146 (76)
Does your unit routinely use parathyroid autofluorescence technology while performing thyroidectomies?	No	157 (81.8)
	Yes	35 (18.2)
Do you routinely use advanced hemostasis devices (e.g., ligasure, harmonic scalpel, etc..) while performing thyroidectomies?	No	28 (14.6)
	Yes	164 (85.4)
Do you routinely use topical hemostatic agents (e.g., tabotamp, tachosil, hemopatch, …) while performing thyroidectomies?	No	101 (52.6)
	Yes	91 (47.4)
Do you believe a standardized definition of “complexity” in thyroid surgery would be useful in stratifying the patient’s baseline risk of postoperative complications and therefore selecting the best workflow to reduce said risk both in open surgery and minimally invasive/remote access surgery?	No	5 (2.6)
	Yes	187 (97.4)

Corrected:

**Table 1:** Respondents’ baseline characteristics

		N (%)
Which surgical specialty are you specialized in?	ENT	4 (2.1)
	General surgery	180 (93.8)
	General surgery, ENT	5 (2.6)
	General surgery, thoracic surgery	3 (1.6)
How long have you been working as a surgeon in your field?	I’m in residency	9 (4.7)
	< 5 years	18 (9.4)
	5–10 years	47 (24.5)
	> 10 years	118 (61.5)
In which of the following setting are you working?	Affiliated private hospital^a^	25 (13)
	Private practice	4 (2.1)
	Public hospital–non teaching	12 (6.3)
	Public hospital–teaching	151 (78.6)
How many total thyroidectomies does your unit perform YEARLY?	< 20	3 (1.6)
	20–49	18 (9.4)
	50–200	69 (35.9)
	> 200	102 (53.1)
How many total thyroidectomies do you personally perform YEARLY?	< 20	21 (10.9)
	20–49	38 (19.8)
	50–200	96 (50)
	> 200	37 (19.3)
Do you personally and routinely use ultrasound to evaluate your patient before surgery?	No	60 (31.3)
	Yes	132 (68.8)
Does your unit routinely use intraoperative nerve monitoring while performing thyroidectomies?	No	46 (24)
	Yes	146 (76)
Does your unit routinely use parathyroid autofluorescence technology while performing thyroidectomies?	No	157 (81.8)
	Yes	35 (18.2)
Do you routinely use advanced hemostasis devices (e.g., ligasure, harmonic scalpel, etc..) while performing thyroidectomies?	No	28 (14.6)
	Yes	164 (85.4)
Do you routinely use topical hemostatic agents (e.g., tabotamp, tachosil, hemopatch, …) while performing thyroidectomies?	No	101 (52.6)
	Yes	91 (47.4)
Do you believe a standardized definition of “complexity” in thyroid surgery would be useful in stratifying the patient’s baseline risk of postoperative complications and therefore selecting the best workflow to reduce said risk both in open surgery and minimally invasive/remote access surgery?	No	5 (2.6)
	Yes	187 (97.4)

*ENT* otolaryngologist, *IONM* intraoperative nerve monitoring, *ICU* intensive care unit

^a^Private hospitals that have an agreement with the national health service (NHS) to provide services that can be reimbursed by the NHS

The original article has been corrected.

