# Supplementary material for: Aligning perspectives: towards a standardized concept of “complexity” in thyroid surgery. An international web-based survey
Source: Updates Surg. 2025 Dec 22;78(2):793–804. doi: 10.1007/s13304-025-02470-0 (PMC13212736; doi:10.1007/s13304-025-02470-0)
Supplement: Supplementary file 1 — Supplementary Material 1. [file 13304_2025_2470_MOESM1_ESM.docx]

| Supplementary Table 1. Comparison between respondents’ answers from units performing >50 thyroidectomies/year and units performing <50 thyroidectomies/year | | | | |
| --- | --- | --- | --- | --- |
|  |  | **<50/year** | **≥50/year** |  |
|  |  | N (%) | N (%) | p-value |
| **Which surgical specialty are you specialized in?** | ENT | 1 (4.8%) | 3 (1.8%) | 0.4 |
|  | General Surgery | 18 (85.7%) | 162 (94.7%) |  |
|  | General Surgery, ENT | 1 (4.8%) | 4 (2.3%) |  |
|  | General Surgery, Thoracic Surgery | 1 (4.8%) | 2 (1.2%) |  |
| **How long have you been working as a surgeon in your field?** | I'm in residency | 3 (14.3%) | 6 (3.5%) | 0.01 |
|  | <5 years | 5 (23.8%) | 13 (7.6%) |  |
|  | 5-10 years | 4 (19%) | 43 (25.1%) |  |
|  | >10 years | 9 (42.9%) | 109 (63.7%) |  |
| **In which of the following setting are you working?** | Affiliated Private Hospital | 3 (14.3%) | 22 (12.9%) | 0.82 |
|  | Private Practice | 1 (4.8%) | 3 (1.8%) |  |
|  | Public Hospital - Non Teaching | 1 (4.8%) | 11 (6.4%) |  |
|  | Public Hospital - Teaching | 16 (76.2%) | 135 (78.9%) |  |
| **How many total thyroidectomies does your unit perform YEARLY?** | <20 | 3 (14.3%) | 0 (0%) | - |
|  | 20-49 | 18 (85.7%) | 0 (0%) |  |
|  | 50-200 | 0 (0%) | 69 (40.4%) |  |
|  | >200 | 0 (0%) | 102 (59.6%) |  |
| **How many total thyroidectomies do you personally perform YEARLY?** | <20 | 8 (38.1%) | 13 (7.6%) | - |
|  | 20-49 | 13 (61.9%) | 25 (14.6%) |  |
|  | 50-200 | 0 (0%) | 96 (56.1%) |  |
|  | >200 | 0 (0%) | 37 (21.6%) |  |
| **Do you personally and routinely use Ultrasound to evaluate your patient before surgery?** | No | 9 (42.9%) | 51 (29.8%) | 0.22 |
|  | Yes | 12 (57.1%) | 120 (70.2%) |  |
| **Does your unit routinely use Intraoperative Nerve Monitoring while performing Thyroidectomies?** | No | 5 (23.8%) | 41 (24%) | 0.99 |
|  | Yes | 16 (76.2%) | 130 (76%) |  |
| **Does your unit routinely use Parathyroid Autofluorescence technology while performing Thyroidectomies?** | No | 16 (76.2%) | 141 (82.5%) | 0.48 |
|  | Yes | 5 (23.8%) | 30 (17.5%) |  |
| **Do you routinely use advanced hemostasis devices (eg. Ligasure, Harmonic scalpel etc..) while performing Thyroidectomies?** | No | 2 (9.5%) | 26 (15.2%) | 0.49 |
|  | Yes | 19 (90.5%) | 145 (84.8%) |  |
| **Do you routinely use topical hemostatic agents (eg. Tabotamp, Tachosil, Hemopatch...) while performing Thyroidectomies?** | No | 11 (52.4%) | 90 (52.6%) | 0.98 |
|  | Yes | 10 (47.6%) | 81 (47.4%) |  |
| **Do you believe a standardized definition of "Complexity" in Thyroid surgery would be useful in stratifying the patient's baseline risk of postoperative complications and therefore selecting the best workflow to reduce said risk both in open surgery and minimally invasive/remote access surgery?** | No | 2 (9.5%) | 3 (1.8%) | 0.04 |
|  | Yes | 19 (90.5%) | 168 (98.2%) |  |
| **Endpoint Measures** |  |  |  |  |
| **Surgery Duration** | No | 8 (38.1%) | 36 (21.1%) | 0.08 |
|  | Yes | 13 (61.9%) | 135 (78.9%) |  |
| **Wound Length** | No | 17 (81%) | 113 (66.1%) | 0.17 |
|  | Yes | 4 (19%) | 58 (33.9%) |  |
| **Postoperative Hematoma Rate** | No | 15 (71.4%) | 93 (54.4%) | 0.14 |
|  | Yes | 6 (28.6%) | 78 (45.6%) |  |
| **Postoperative Transient Vocal Cord Palsy rate** | No | 9 (42.9%) | 51 (29.8%) | 0.22 |
|  | Yes | 12 (57.1%) | 120 (70.2%) |  |
| **Postoperative Permanent Vocal Cord Palsy rate** | No | 8 (38.1%) | 33 (19.3%) | 0.05 |
|  | Yes | 13 (61.9%) | 138 (80.7%) |  |
| **Postoperative Transient Hypoparathyroidism rate** | No | 10 (47.6%) | 63 (36.8%) | 0.34 |
|  | Yes | 11 (52.4%) | 108 (63.2%) |  |
| **Postoperative Permanent Hypoparathyroidism rate** | No | 7 (33.3%) | 37 (21.6%) | 0.23 |
|  | Yes | 14 (66.7%) | 134 (78.4%) |  |
| **Tracheal Injury rate** | No | 16 (76.2%) | 88 (51.5%) | 0.03 |
|  | Yes | 5 (23.8%) | 83 (48.5%) |  |
| **R1 Resection Rate (if Thyroid Neoplasm is involved)** | No | 14 (66.7%) | 73 (42.7%) | 0.04 |
|  | Yes | 7 (33.3%) | 98 (57.3%) |  |
| **Recurrence Rate (if Thyroid Neoplasm/Basedow disease is involved)** | No | 15 (71.4%) | 101 (59.1%) | 0.27 |
|  | Yes | 6 (28.6%) | 70 (40.9%) |  |
| **Intraoperative Mortality rate** | No | 16 (76.2%) | 140 (81.9%) | 0.53 |
|  | Yes | 5 (23.8%) | 31 (18.1%) |  |
| **Postoperative Mortality rate** | No | 13 (61.9%) | 131 (76.6%) | 0.14 |
|  | Yes | 8 (38.1%) | 40 (23.4%) |  |
| **Surgical Site Infection rate** | No | 17 (81%) | 130 (76%) | 0.62 |
|  | Yes | 4 (19%) | 41 (24%) |  |
| **Anesthesia-related Complication rate** | No | 17 (81%) | 129 (75.4%) | 0.58 |
|  | Yes | 4 (19%) | 42 (24.6%) |  |
| **Postoperative ICU admission rate** | No | 14 (66.7%) | 111 (64.9%) | 0.87 |
|  | Yes | 7 (33.3%) | 60 (35.1%) |  |
| **Postoperative Length of Hospital Stay** | No | 11 (52.4%) | 89 (52%) | 0.98 |
|  | Yes | 10 (47.6%) | 82 (48%) |  |
| **Readmission rate** | No | 16 (76.2%) | 112 (65.5%) | 0.33 |
|  | Yes | 5 (23.8%) | 59 (34.5%) |  |
| **New complications rate** | No | 21 (100%) | 171 (100%) |  |
| ENT, Otolaryngologyst; IONM, Intraoperative Nerve Monitoring; ICU, Intensive Care Unit. | | | | |

| Supplementary Table 2. Comparison between respondents’ answers from surgeons performing >50 thyroidectomies/year and surgeons performing <50 thyroidectomies/year | | | | |
| --- | --- | --- | --- | --- |
|  |  | **<50/year** | **≥50/year** |  |
|  |  | N (%) | N (%) | p-value |
| **Which surgical specialty are you specialized in?** | ENT | 1 (1.7%) | 3 (2.3%) | 0.55 |
|  | General Surgery | 54 (91.5%) | 126 (94.7%) |  |
|  | General Surgery, ENT | 3 (5.1%) | 2 (1.5%) |  |
|  | General Surgery, Thoracic Surgery | 1 (1.7%) | 2 (1.5%) |  |
| **How long have you been working as a surgeon in your field?** | I'm in residency | 9 (15.3%) | 0 (0%) | <0.0005 |
|  | <5 years | 9 (15.3%) | 9 (6.8%) |  |
|  | 5-10 years | 15 (25.4%) | 32 (24.1%) |  |
|  | >10 years | 26 (44.1%) | 92 (69.2%) |  |
| **In which of the following setting are you working?** | Affiliated Private Hospital | 5 (8.5%) | 20 (15%) | 0.57 |
|  | Private Practice | 1 (1.7%) | 3 (2.3%) |  |
|  | Public Hospital - Non Teaching | 3 (5.1%) | 9 (6.8%) |  |
|  | Public Hospital - Teaching | 50 (84.7%) | 101 (75.9%) |  |
| **How many total thyroidectomies does your unit perform YEARLY?** | <20 | 3 (5.1%) | 0 (0%) | <0.0005 |
|  | 20-50 | 18 (30.5%) | 0 (0%) |  |
|  | 50-200 | 24 (40.7%) | 45 (33.8%) |  |
|  | >200 | 14 (23.7%) | 88 (66.2%) |  |
| **How many total thyroidectomies do you personally perform YEARLY?** | <20 | 21 (35.6%) | 0 (0%) | - |
|  | >200 | 38 (64.4%) | 0 (0%) |  |
|  | 20-50 | 0 (0%) | 96 (72.2%) |  |
|  | 50-200 | 0 (0%) | 37 (27.8%) |  |
| **Do you personally and routinely use Ultrasound to evaluate your patient before surgery?** | No | 17 (28.8%) | 43 (32.3%) | 0.63 |
|  | Yes | 42 (71.2%) | 90 (67.7%) |  |
| **Does your unit routinely use Intraoperative Nerve Monitoring while performing Thyroidectomies?** | No | 21 (35.6%) | 25 (18.8%) | 0.01 |
|  | Yes | 38 (64.4%) | 108 (81.2%) |  |
| **Does your unit routinely use Parathyroid Autofluorescence technology while performing Thyroidectomies?** | No | 50 (84.7%) | 107 (80.5%) | 0.48 |
|  | Yes | 9 (15.3%) | 26 (19.5%) |  |
| **Do you routinely use advanced hemostasis devices (eg. Ligasure, Harmonic scalpel etc..) while performing Thyroidectomies?** | No | 12 (20.3%) | 16 (12%) | 0.13 |
|  | Yes | 47 (79.7%) | 117 (88%) |  |
| **Do you routinely use topical hemostatic agents (eg. Tabotamp, Tachosil, Hemopatch...) while performing Thyroidectomies?** | No | 24 (40.7%) | 77 (57.9%) | 0.03 |
|  | Yes | 35 (59.3%) | 56 (42.1%) |  |
| **Do you believe a standardized definition of "Complexity" in Thyroid surgery would be useful in stratifying the patient's baseline risk of postoperative complications and therefore selecting the best workflow to reduce said risk both in open surgery and minimally invasive/remote access surgery?** | No | 3 (5.1%) | 2 (1.5%) | 0.15 |
|  | Yes | 56 (94.9%) | 131 (98.5%) |  |
| **Endpoint measures** |  |  |  |  |
| **Surgery Duration** | No | 17 (28.8%) | 27 (20.3%) | 0.2 |
|  | Yes | 42 (71.2%) | 106 (79.7%) |  |
| **Wound Length** | No | 46 (78%) | 84 (63.2%) | 0.04 |
|  | Yes | 13 (22%) | 49 (36.8%) |  |
| **Postoperative Hematoma Rate** | No | 33 (55.9%) | 75 (56.4%) | 0.95 |
|  | Yes | 26 (44.1%) | 58 (43.6%) |  |
| **Postoperative Transient Vocal Cord Palsy rate** | No | 20 (33.9%) | 40 (30.1%) | 0.6 |
|  | Yes | 39 (66.1%) | 93 (69.9%) |  |
| **Postoperative Permanent Vocal Cord Palsy rate** | No | 16 (27.1%) | 25 (18.8%) | 0.19 |
|  | Yes | 43 (72.9%) | 108 (81.2%) |  |
| **Postoperative Transient Hypoparathyroidism rate** | No | 23 (39%) | 50 (37.6%) | 0.85 |
|  | Yes | 36 (61%) | 83 (62.4%) |  |
| **Postoperative Permanent Hypoparathyroidism rate** | No | 16 (27.1%) | 28 (21.1%) | 0.36 |
|  | Yes | 43 (72.9%) | 105 (78.9%) |  |
| **Tracheal Injury rate** | No | 34 (57.6%) | 70 (52.6%) | 0.52 |
|  | Yes | 25 (42.4%) | 63 (47.4%) |  |
| **R1 Resection Rate (if Thyroid Neoplasm is involved)** | No | 33 (55.9%) | 54 (40.6%) | 0.05 |
|  | Yes | 26 (44.1%) | 79 (59.4%) |  |
| **Recurrence Rate (if Thyroid Neoplasm/Basedow disease is involved)** | No | 41 (69.5%) | 75 (56.4%) | 0.09 |
|  | Yes | 18 (30.5%) | 58 (43.6%) |  |
| **Intraoperative Mortality rate** | No | 45 (76.3%) | 111 (83.5%) | 0.24 |
|  | Yes | 14 (23.7%) | 22 (16.5%) |  |
| **Postoperative Mortality rate** | No | 40 (67.8%) | 104 (78.2%) | 0.12 |
|  | Yes | 19 (32.2%) | 29 (21.8%) |  |
| **Surgical Site Infection rate** | No | 45 (76.3%) | 102 (76.7%) | 0.95 |
|  | Yes | 14 (23.7%) | 31 (23.3%) |  |
| **Anesthesia-related Complication rate** | No | 47 (79.7%) | 99 (74.4%) | 0.43 |
|  | Yes | 12 (20.3%) | 34 (25.6%) |  |
| **Postoperative ICU admission rate** | No | 40 (67.8%) | 85 (63.9%) | 0.6 |
|  | Yes | 19 (32.2%) | 48 (36.1%) |  |
| **Postoperative Length of Hospital Stay** | No | 33 (55.9%) | 67 (50.4%) | 0.48 |
|  | Yes | 26 (44.1%) | 66 (49.6%) |  |
| **Readmission rate** | No | 42 (71.2%) | 86 (64.7%) | 0.38 |
|  | Yes | 17 (28.8%) | 47 (35.3%) |  |
| **New complications rate** | No | 59 (100%) | 133 (100%) |  |
| ENT, Otolaryngologyst; IONM, Intraoperative Nerve Monitoring; ICU, Intensive Care Unit. | | | | |

| Supplementary Table 3. Comparison of respondents’ answers between non routine US users and routine US users | | | | |
| --- | --- | --- | --- | --- |
|  |  | **Non-US users** | **US users** |  |
|  |  | N (%) | N (%) | p-value |
| **Which surgical specialty are you specialized in?** | ENT | 1 (1.7%) | 3 (2.3%) | 0.07 |
|  | General Surgery | 55 (91.7%) | 125 (94.7%) |  |
|  | General Surgery, ENT | 1 (1.7%) | 4 (3%) |  |
|  | General Surgery, Thoracic Surgery | 3 (5%) | 0 (0%) |  |
| **How long have you been working as a surgeon in your field?** | I'm in residency | 3 (5%) | 6 (4.5%) | 0.06 |
|  | <5 years | 9 (15%) | 9 (6.8%) |  |
|  | 5-10 years | 19 (31.7%) | 28 (21.2%) |  |
|  | >10 years | 29 (48.3%) | 89 (67.4%) |  |
| **In which of the following setting are you working?** | Affiliated Private Hospital | 9 (15%) | 16 (12.1%) | 0.11 |
|  | Private Practice | 1 (1.7%) | 3 (2.3%) |  |
|  | Public Hospital - Non Teaching | 0 (0%) | 12 (9.1%) |  |
|  | Public Hospital - Teaching | 50 (83.3%) | 101 (76.5%) |  |
| **How many total thyroidectomies does your unit perform YEARLY?** | <20 | 1 (1.7%) | 2 (1.5%) | 0.16 |
|  | 20-50 | 8 (13.3%) | 10 (7.6%) |  |
|  | 50-200 | 15 (25%) | 54 (40.9%) |  |
|  | >200 | 36 (60%) | 66 (50%) |  |
| **How many total thyroidectomies do you personally perform YEARLY?** | <20 | 5 (8.3%) | 16 (12.1%) | 0.54 |
|  | 20-50 | 12 (20%) | 26 (19.7%) |  |
|  | 50-200 | 34 (56.7%) | 62 (47%) |  |
|  | >200 | 9 (15%) | 28 (21.2%) |  |
| **Do you personally and routinely use Ultrasound to evaluate your patient before surgery?** | No | 60 (100%) | 0 (0%) | - |
|  | Yes | 0 (0%) | 132 (100%) |  |
| **Does your unit routinely use Intraoperative Nerve Monitoring while performing Thyroidectomies?** | No | 11 (18.3%) | 35 (26.5%) | 0.2 |
|  | Yes | 49 (81.7%) | 97 (73.5%) |  |
| **Does your unit routinely use Parathyroid Autofluorescence technology while performing Thyroidectomies?** | No | 48 (80%) | 109 (82.6%) | 0.67 |
|  | Yes | 12 (20%) | 23 (17.4%) |  |
| **Do you routinely use advanced hemostasis devices (eg. Ligasure, Harmonic scalpel etc..) while performing Thyroidectomies?** | No | 5 (8.3%) | 23 (17.4%) | 0.1 |
|  | Yes | 55 (91.7%) | 109 (82.6%) |  |
| **Do you routinely use topical hemostatic agents (eg. Tabotamp, Tachosil, Hemopatch...) while performing Thyroidectomies?** | No | 35 (58.3%) | 66 (50%) | 0.28 |
|  | Yes | 25 (41.7%) | 66 (50%) |  |
| **Do you believe a standardized definition of "Complexity" in Thyroid surgery would be useful in stratifying the patient's baseline risk of postoperative complications and therefore selecting the best workflow to reduce said risk both in open surgery and minimally invasive/remote access surgery?** | No | 0 (0%) | 5 (3.8%) | 0.13 |
|  | Yes | 60 (100%) | 127 (96.2%) |  |
| **Endpoint measures** |  |  |  |  |
| **Surgery Duration** | No | 13 (21.7%) | 31 (23.5%) | 0.78 |
|  | Yes | 47 (78.3%) | 101 (76.5%) |  |
| **Wound Length** | No | 39 (65%) | 91 (68.9%) | 0.59 |
|  | Yes | 21 (35%) | 41 (31.1%) |  |
| **Postoperative Hematoma Rate** | No | 33 (55%) | 75 (56.8%) | 0.81 |
|  | Yes | 27 (45%) | 57 (43.2%) |  |
| **Postoperative Transient Vocal Cord Palsy rate** | No | 20 (33.3%) | 40 (30.3%) | 0.67 |
|  | Yes | 40 (66.7%) | 92 (69.7%) |  |
| **Postoperative Permanent Vocal Cord Palsy rate** | No | 13 (21.7%) | 28 (21.2%) | 0.94 |
|  | Yes | 47 (78.3%) | 104 (78.8%) |  |
| **Postoperative Transient Hypoparathyroidism rate** | No | 26 (43.3%) | 47 (35.6%) | 0.31 |
|  | Yes | 34 (56.7%) | 85 (64.4%) |  |
| **Postoperative Permanent Hypoparathyroidism rate** | No | 14 (23.3%) | 30 (22.7%) | 0.93 |
|  | Yes | 46 (76.7%) | 102 (77.3%) |  |
| **Tracheal Injury rate** | No | 29 (48.3%) | 75 (56.8%) | 0.27 |
|  | Yes | 31 (51.7%) | 57 (43.2%) |  |
| **R1 Resection Rate (if Thyroid Neoplasm is involved)** | No | 23 (38.3%) | 64 (48.5%) | 0.19 |
|  | Yes | 37 (61.7%) | 68 (51.5%) |  |
| **Recurrence Rate (if Thyroid Neoplasm/Basedow disease is involved)** | No | 31 (51.7%) | 85 (64.4%) | 0.09 |
|  | Yes | 29 (48.3%) | 47 (35.6%) |  |
| **Intraoperative Mortality rate** | No | 48 (80%) | 108 (81.8%) | 0.76 |
|  | Yes | 12 (20%) | 24 (18.2%) |  |
| **Postoperative Mortality rate** | No | 46 (76.7%) | 98 (74.2%) | 0.72 |
|  | Yes | 14 (23.3%) | 34 (25.8%) |  |
| **Surgical Site Infection rate** | No | 43 (71.7%) | 104 (78.8%) | 0.28 |
|  | Yes | 17 (28.3%) | 28 (21.2%) |  |
| **Anesthesia-related Complication rate** | No | 50 (83.3%) | 96 (72.7%) | 0.11 |
|  | Yes | 10 (16.7%) | 36 (27.3%) |  |
| **Postoperative ICU admission rate** | No | 42 (70%) | 83 (62.9%) | 0.34 |
|  | Yes | 18 (30%) | 49 (37.1%) |  |
| **Postoperative Length of Hospital Stay** | No | 36 (60%) | 64 (48.5%) | 0.14 |
|  | Yes | 24 (40%) | 68 (51.5%) |  |
| **Readmission rate** | No | 41 (68.3%) | 87 (65.9%) | 0.74 |
|  | Yes | 19 (31.7%) | 45 (34.1%) |  |
| **New complications rate** | No | 60 (100%) | 132 (100%) |  |
| ENT, Otolaryngologyst; IONM, Intraoperative Nerve Monitoring; ICU, Intensive Care Unit. | | | | |

| Supplementary Table 4. Comparison of respondents’ answers between non routine IONM users and routine IONM users | | | | |
| --- | --- | --- | --- | --- |
|  |  | **Non-IONM users** | **IONM users** |  |
|  |  | N (%) | N (%) | p-value |
| **Which surgical specialty are you specialized in?** | ENT | 2 (4.3%) | 2 (1.4%) | 0.36 |
|  | General Surgery | 43 (93.5%) | 137 (93.8%) |  |
|  | General Surgery, ENT | 0 (0%) | 5 (3.4%) |  |
|  | General Surgery, Thoracic Surgery | 1 (2.2%) | 2 (1.4%) |  |
| **How long have you been working as a surgeon in your field?** | I'm in residency | 4 (8.7%) | 5 (3.4%) | 0.36 |
|  | <5 years | 3 (6.5%) | 15 (10.3%) |  |
|  | 5-10 years | 13 (28.3%) | 34 (23.3%) |  |
|  | >10 years | 26 (56.5%) | 92 (63%) |  |
| **In which of the following setting are you working?** | Affiliated Private Hospital | 5 (10.9%) | 20 (13.7%) | 0.11 |
|  | Private Practice | 0 (0%) | 4 (2.7%) |  |
|  | Public Hospital - Non Teaching | 6 (13%) | 6 (4.1%) |  |
|  | Public Hospital - Teaching | 35 (76.1%) | 116 (79.5%) |  |
| **How many total thyroidectomies does your unit perform YEARLY?** | <20 | 2 (4.3%) | 1 (0.7%) | 0.16 |
|  | 20-49 | 3 (6.5%) | 15 (10.3%) |  |
|  | 50-200 | 20 (43.5%) | 49 (33.6%) |  |
|  | >200 | 21 (45.7%) | 81 (55.5%) |  |
| **How many total thyroidectomies do you personally perform YEARLY?** | <20 | 9 (19.6%) | 12 (8.2%) | 0.03 |
|  | 20-49 | 12 (26.1%) | 26 (17.8%) |  |
|  | 50-200 | 21 (45.7%) | 75 (51.4%) |  |
|  | >200 | 4 (8.7%) | 33 (22.6%) |  |
| **Do you personally and routinely use Ultrasound to evaluate your patient before surgery?** | No | 11 (23.9%) | 49 (33.6%) | 0.22 |
|  | Yes | 35 (76.1%) | 97 (66.4%) |  |
| **Does your unit routinely use Intraoperative Nerve Monitoring while performing Thyroidectomies?** | No | 46 (100%) | 0 (0%) |  |
|  | Yes | 0 (0%) | 146 (100%) |  |
| **Does your unit routinely use Parathyroid Autofluorescence technology while performing Thyroidectomies?** | No | 43 (93.5%) | 114 (78.1%) | 0.02 |
|  | Yes | 3 (6.5%) | 32 (21.9%) |  |
| **Do you routinely use advanced hemostasis devices (eg. Ligasure, Harmonic scalpel etc..) while performing Thyroidectomies?** | No | 11 (23.9%) | 17 (11.6%) | 0.04 |
|  | Yes | 35 (76.1%) | 129 (88.4%) |  |
| **Do you routinely use topical hemostatic agents (eg. Tabotamp, Tachosil, Hemopatch...) while performing Thyroidectomies?** | No | 23 (50%) | 78 (53.4%) | 0.69 |
|  | Yes | 23 (50%) | 68 (46.6%) |  |
| **Do you believe a standardized definition of "Complexity" in Thyroid surgery would be useful in stratifying the patient's baseline risk of postoperative complications and therefore selecting the best workflow to reduce said risk both in open surgery and minimally invasive/remote access surgery?** | No | 1 (2.2%) | 4 (2.7%) | 0.83 |
|  | Yes | 45 (97.8%) | 142 (97.3%) |  |
| **Endpoint Measures** |  |  |  |  |
| **Surgery Duration** | No | 14 (30.4%) | 30 (20.5%) | 0.16 |
|  | Yes | 32 (69.6%) | 116 (79.5%) |  |
| **Wound Length** | No | 30 (65.2%) | 100 (68.5%) | 0.68 |
|  | Yes | 16 (34.8%) | 46 (31.5%) |  |
| **Postoperative Hematoma Rate** | No | 27 (58.7%) | 81 (55.5%) | 0.7 |
|  | Yes | 19 (41.3%) | 65 (44.5%) |  |
| **Postoperative Transient Vocal Cord Palsy rate** | No | 14 (30.4%) | 46 (31.5%) | 0.89 |
|  | Yes | 32 (69.6%) | 100 (68.5%) |  |
| **Postoperative Permanent Vocal Cord Palsy rate** | No | 15 (32.6%) | 26 (17.8%) | 0.03 |
|  | Yes | 31 (67.4%) | 120 (82.2%) |  |
| **Postoperative Transient Hypoparathyroidism rate** | No | 17 (37%) | 56 (38.4%) | 0.86 |
|  | Yes | 29 (63%) | 90 (61.6%) |  |
| **Postoperative Permanent Hypoparathyroidism rate** | No | 16 (34.8%) | 28 (19.2%) | 0.03 |
|  | Yes | 30 (65.2%) | 118 (80.8%) |  |
| **Tracheal Injury rate** | No | 24 (52.2%) | 80 (54.8%) | 0.76 |
|  | Yes | 22 (47.8%) | 66 (45.2%) |  |
| **R1 Resection Rate (if Thyroid Neoplasm is involved)** | No | 26 (56.5%) | 61 (41.8%) | 0.08 |
|  | Yes | 20 (43.5%) | 85 (58.2%) |  |
| **Recurrence Rate (if Thyroid Neoplasm/Basedow disease is involved)** | No | 29 (63%) | 87 (59.6%) | 0.68 |
|  | Yes | 17 (37%) | 59 (40.4%) |  |
| **Intraoperative Mortality rate** | No | 35 (76.1%) | 121 (82.9%) | 0.3 |
|  | Yes | 11 (23.9%) | 25 (17.1%) |  |
| **Postoperative Mortality rate** | No | 36 (78.3%) | 108 (74%) | 0.56 |
|  | Yes | 10 (21.7%) | 38 (26%) |  |
| **Surgical Site Infection rate** | No | 37 (80.4%) | 110 (75.3%) | 0.48 |
|  | Yes | 9 (19.6%) | 36 (24.7%) |  |
| **Anesthesia-related Complication rate** | No | 34 (73.9%) | 112 (76.7%) | 0.7 |
|  | Yes | 12 (26.1%) | 34 (23.3%) |  |
| **Postoperative ICU admission rate** | No | 35 (76.1%) | 90 (61.6%) | 0.07 |
|  | Yes | 11 (23.9%) | 56 (38.4%) |  |
| **Postoperative Length of Hospital Stay** | No | 28 (60.9%) | 72 (49.3%) | 0.17 |
|  | Yes | 18 (39.1%) | 74 (50.7%) |  |
| **Readmission rate** | No | 35 (76.1%) | 93 (63.7%) | 0.12 |
|  | Yes | 11 (23.9%) | 53 (36.3%) |  |
| **New complications rate** | No | 46 (100%) | 146 (100%) |  |
| ENT, Otolaryngologyst; IONM, Intraoperative Nerve Monitoring; ICU, Intensive Care Unit. | | | | |
